# Prevalence of Carcinogenic Genotypes of HPV-Infected Women in a Ten-Year Period (2014–2023) in Vojvodina, Serbia

**DOI:** 10.3390/medicina60060922

**Published:** 2024-06-01

**Authors:** Natasa Nikolic, Branka Basica, Mirjana Strbac, Lidija Terzic, Aleksandra Patic, Gordana Kovacevic, Radmila Velicki, Dusan Petrovic, Aljosa Mandic, Vladimir Petrovic

**Affiliations:** 1 Institute of Public Health of Vojvodina, 21000 Novi Sad, Serbia; natasa.nikolic@mf.uns.ac.rs (N.N.); mirjana.strbac@izjzv.org.rs (M.S.); lidija.terzic@izjzv.org.rs (L.T.); aleksandra.patic@mf.uns.ac.rs (A.P.); gordana.kovacevic@izjzv.org.rs (G.K.); radmila.velicki@mf.uns.ac.rs (R.V.); dusan.petrovic@izjzv.org.rs (D.P.); vladimir.petrovic@mf.uns.ac.rs (V.P.); 2 Faculty of Medicine, University of Novi Sad, 21000 Novi Sad, Serbia; aljosa.mandic@mf.uns.ac.rs; 3 Clinic for Oncological Surgery, Oncology Institute of Vojvodina, 21204 Sremska Kamenica, Serbia

**Keywords:** HPV, prevalence, genotype distribution, cervical intraepithelial lesion, Serbia

## Abstract

*Background and Objectives*: Human papillomavirus (HPV) infection and its etiological role in the development of cervical cancer are well established. The cervical cancer mortality rate in Serbia is one of the highest among European countries, and this cancer is the second-leading cause of death in Serbian women aged from 15 to 44. *Materials and Methods*: This retrospective study was conducted at the Institute of Public Health of Vojvodina. A total of 10,062 cervical specimens from Serbian women were collected and HPV tested in ten years. The study patients were divided into five age groups. HPV genotype testing was performed using a commercial kit to detect 14 high-risk (HR) HPV genotypes. Additionally, cervix cytology data have been available for patients tested in 2022 and 2023. *Results*: An overall positive rate was found in 43.3% of patients (4356/10,062). A single HPV infection (62.1%) was the main infection pattern. The most frequent HR HPV genotypes were HPV 16, 31, 52, 56, 39, and 51, comprising 62.3% of the detected genotypes, including multiple infections. A significant difference was noted in the HPV prevalence across the different age groups, with a bimodal distribution of HPV infection. The highest prevalence was recorded in the age group ≤ 30 and those after 61 years. Women diagnosed with high-grade squamous intraepithelial lesions (HSIL) were significantly older compared to others. HR HPV is the most prevalent in patients with HSIL cytological findings (76.5%). The most common type, according to age-specific distribution and cytological findings, was HR HPV 16. *Conclusions*: This study provides comprehensive data on HR HPV distribution among Serbian women, which can serve as a basis for subsequent monitoring of genotypic distribution. It is particularly significant considering they are missing in the updated ICO/IARC Report for Serbia, and the cervical cancer mortality rate in Serbia is one of the highest among European countries.

## 1. Introduction

Human papillomaviruses (HPVs) are small, double-stranded epitheliotropic DNA viruses. Now it is convincingly established that HPV infection is the causal association with cervical cancer, and growing evidence indicates that HPV is a relevant factor in developing malignant diseases of different locations (vulva, vagina, penis, anus, and head and neck) [1]. Cervical cancer is one of the most common malignant tumours in women and is the leading cause of cancer death in 36 countries [2]. It has the fourth-highest incidence and mortality rates among women worldwide, with 604,000 new cases and 342,000 deaths in 2020 [3]. Persistent HPV infection is associated with an extremely high absolute risk of cancer and is a necessary condition for cervical neoplasia [4]. It is estimated that the genital tract can be infected with about 40 genotypes of the Alpha genus HPV, but not all have been linked to cervical cancer. According to their carcinogenicity, alpha HPVs are classified as high-risk HPV (HR HPV) and low-risk HPV (LR HPV) as follows [4]:

Group 1 (carcinogenic to humans, HR) includes HPV genotypes 16, 18, 31, 33, 35, 39, 45, 51, 52, 56, 58, and 59;
Group 2A (probably carcinogenic) includes HPV genotype 68;
Group 2B (potentially carcinogenic) includes HPV genotypes 26, 53, 66, 67, 70, 73, 82, 30, 34, 69, 85, and 97;
Group 3 (low-risk, LR) includes HPV genotypes 6 and 11.

More precisely, it has been proven that HR HPV 16 and 18 are the most virulent HPV genotypes and cause 70% of cervical cancer cases [3], while, together with HR HPV 31, 33, 45, 52, and 58, they are the cause of about 90% of all cervical cancer cases [5]. It can be concluded that although HPV infections worldwide are caused mainly by the same high-risk HPV genotypes, there is variability in their epidemiologic distribution. Morbidity and mortality-HPV-associated factors include geographic, socioeconomic, cultural, and HPV genome variability factors and intrinsic characteristics of HPV-infected individuals [6].

Nowadays, infection caused by HPV is one of the most common sexually transmitted infections, and, according to estimates, over 75% of sexually active women are infected with this virus at some point in their lives. In most cases, the infection will be transient and resolved by the immune system within 24 months (reviewed in [1]). This initial infection proceeds without developing a clinically manifest disease [1], and the peak of its specific incidence rate is registered in the early twenties of life. However, natural HPV infection rarely persists, and over 90% of detectable infections are cleared by cellular immunity and not detected within five to seven years [7,8,9,10]. Persistent infection leads to the formation of cervical intraepithelial neoplasia of varying degrees, which can eventually develop into cancer [4].

Vaccination against HPV is one of the most effective tools for HPV-related precancers and cancer prevention. In general, the implementation of HPV vaccines in public health strategies has resulted in a decrease in the prevalence of vaccine-targeted HPV infection as well as markedly impacted HPV-related disease incidence, such as warts and precancerous lesions [11]. There are three currently available prophylactic HPV vaccines: the bivalent (2vHPV), which consists of virus-like particles against HR HPV 16 and 18, and the quadrivalent (4vHPV) vaccine, which also protects against HR HPV 16 and 18 but also against HPV 6 and 11; while 9-valent (9vHPV) vaccines, next to the mentioned genotypes, protect against HR HPV 31, 33, 45, 52, and 58 [12]. However, complete protection against all oncogenic HPVs using these vaccines has not been provided; therefore, cervical screening remains an indispensable preventive measure [11]. It has been proven that a significant impact in reducing the prevalence of cervical cancer has been achieved with the wide use of the Papanicolaou (PAP) test based on cytomorphological examination [13,14]. The knowledge gained in the last few decades has undoubtedly pointed to the etiological role of HPV in the oncogenesis of cervical cancer, which led to improvement in laboratory diagnostics of this disease and the introduction of HPV molecular screening tests. HPV testing is based on detecting HPV DNA, mRNA, or other viral biomarkers. HPV DNA tests are more sensitive and accurate than cytology, indicating that this testing has become an essential part of guidelines for cervical carcinoma screening and follow-up (reviewed in [14]).

In Serbia, organised cervical screening has been conducted since 2012, and the target group is women aged from 25 to 65. HPV vaccines have been available and recommended since 2018 in Serbia. In 2022, the 9vHPV vaccine was introduced in the National Immunisation Program in Serbia and applied nationwide free of charge [15].

However, the incidence of cervical cancer in Serbia is still among the highest. It is approximately twice the average in Europe (10.7 to 100,000), and cervical cancer is the second most common cancer in women aged from 15 to 44 [3,16]. Besides that, it is essential to note that HPV prevalence and genotype distribution of normal and abnormal cervical lesions are missing in the updated IARC Human Papillomavirus and Related Diseases Report for Serbia [16]. Additionally, recently published data from other regions of the world (reviewed in [6]) indicate the importance of conducting this type of research. Therefore, we aimed to investigate the prevalence and diversity of HPV infections in Serbian women and further evaluate the genotype- and age-specific distribution with different cytological groups. Knowledge of local HPV prevalence and genotypic distribution is necessary for successfully establishing disease prevention and control measures, that is, their implementation in the national strategy.

## 2. Materials and Methods

### 2.1. Study Subjects

From 2014 to 2023, cervical smears were obtained from a sample of 10,062 female patients over 18 years old undergoing gynaecological exams at the Department of Gynaecology, Community Health Centre Novi Sad, Serbia. The samples were collected and sent to the Centre of Virology, Institute of Public Health of Vojvodina, Novi Sad, Republic of Serbia, for further analyses. This retrospective study was conducted at the same institute. The screened patients in this comprehensive study ranged from 18 to 84 years old, with a mean age of 38.07 ± 10.98 years. To ensure a representative sample across different age groups, they were thoughtfully divided into five groups: ≤30, 31–40, 41–50, 51–60, and ≥61 years. Additional cervical intraepithelial cytology data are available for patients tested from 2022 to 2023 (total of 2026 women). Cytology findings included in the analysis, acquired through conventional PAP smear testing, were based on the criteria of the Bethesda System 2014 [17]. These were categorised into negative, for an intraepithelial lesion or malignancy (NILM), atypical squamous cells of unknown significance (ASCUS), low-grade squamous intraepithelial lesions (LSILs), and high-grade squamous intraepithelial lesions (HSILs). Written patient consent was unnecessary because the laboratory service request accompanies each cervical sample form, which has to be signed and approved by the gynaecologist responsible for the verbal patient consent obtained for each cervical specimen collected for HPV diagnosis. The utilisation of medical documentation received approval from the Medical Ethical Committee of the Institute of Public Health of Vojvodina (approval number: 01-641/2). The handling and publication of patients’ data in this study were strictly in accordance with the Declaration of Helsinki, including confidentiality and anonymity.

### 2.2. HR HPV Detection and Genotyping

The cervical smear samples were stored at 4–8 °C for up to 3 days from the sampling day. According to the manufacturer’s instructions, DNA extraction was performed using the SaMag STD DNA Extraction Kit (Sacace Biotechnologies, Como, Italy). The extracted DNA was eluted in 100 µL elution buffer. The samples collected during 2014, 2019, 2020, and 2021 were tested using the HPV High-Risk Typing Real-TM Kit (Sacace Biotechnologies, Como, Italy), which detects and genotypes twelve HR HPVs (16, 18, 31, 33, 35, 39, 45, 51, 52, 56, 58, and 59). A detailed protocol was previously described by Nikolic et al. (2023) [18]. Samples collected during the other six years were tested using the HPV Genotypes 14 Real-TM Quant Real-Time PCR Kit (Sacace Biotechnologies, Como, Italy) to detect and genotype 14 HR HPVs (16, 18, 31, 33, 35, 39, 45, 51, 52, 56, 58, 59, 66, and 68). E1 gene (HPV 56), E6 gene (HPV 16, 31, 33, 45, 59, 66, and 68), E6/E7 gene (HPV 35), and E7 gene (HPV 18, 39, 51, 52, and 58) genotypes were amplified using primers and TaqMan probes in the four separate multiplex reactions each performed in a total volume of 25 µL. The PCR reactions included reaction quality controls: positive control, negative control, and internal quality control (β globin gene) that ensured the cervical epithelial cells were adequately collected. Real-time PCR was performed on the SaCycler-96 (Sacace Biotechnologies, Como, Italy) or the QuantStudio 5 Real-Time PCR System (Thermo Fisher Scientific, Waltham, MA, USA). After the initial activation of the DNA polymerase at 95 °C for 15 min, five cycles of amplification were performed under the following conditions: 95 °C/5 s, 60 °C/20 s, and 72 °C/15 s, and 40 amplifications were performed under the following conditions: 95 °C/5 s, 60 °C/30 s (fluorescence signal detection), and 72 °C/15 s. The kinetics of the detected fluorescence signals were monitored using the SaCycler-96 software package (v7.3) (Sacace Biotechnologies, Como, Italy) or QuantStudio 5 Real-Time PCR software (v1.5.2) (Thermo Fisher Scientific Waltham, MA, USA). The data were analysed using GraphPad Prism 8 (GraphPad Software, San Diego, CA, USA).

### 2.3. Statistical Analysis

Data were collected in Microsoft Excel (2019) tables, and statistical analyses were performed using SPSS statistics software Version 21.0 (Chicago, IL, USA). The HPV genotype distribution was depicted through metrics such as infection rate and prevalence, expressed as a percentage. Testing the difference in frequencies of attributive features, prevalence, genotype, and number of co-infections of HR HPV in different age and cytological groups was performed using the Chi-square (χ^2^) test of independence and quality of the match. A one-way analysis of the variance (ANOVA) and the Bonferroni post-hoc test were applied to compare age, as the only continuous variable. A *p*-value below 0.05 was used as the threshold for determining statistical significance for all statistical analyses.

## 3. Results

### 3.1. Overall HR HPV Infection Prevalence

**Figure 1:**
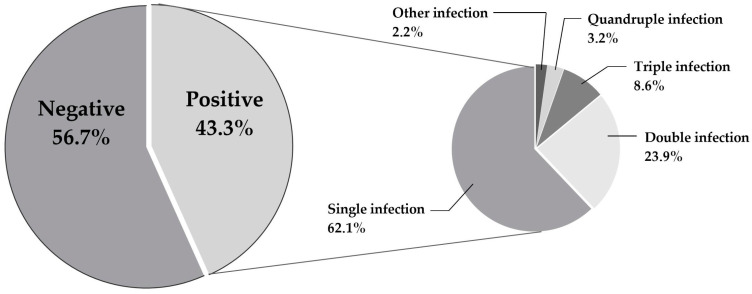
Overall HR HPV prevalence with the ratio of HR HPV infection.

In this study, 10,062 samples from female patients were collected between 2014 and 2023, among which 4356 women were screened positive for HR HPV, which indicates that the overall prevalence of HPV infection in the study population was 43.3% (Figure 1). Furthermore, among these infections, 2705 (62.1%) women were positive for one HR HPV genotype (single infection), while 1651 women were positive for infections caused by more than one HR HPV genotype, including 1040 (23.9%) patients with double infections, 375 (8.6%) patients with triple infections, 138 (3.2%) patients with quadruple infections, and 98 (2.2%) patients with more than four detected HR HPV genotypes in cervical sample (Figure 1).

### 3.2. Genotype-Specific Prevalence of HR HPV Infection

**Figure 2:**
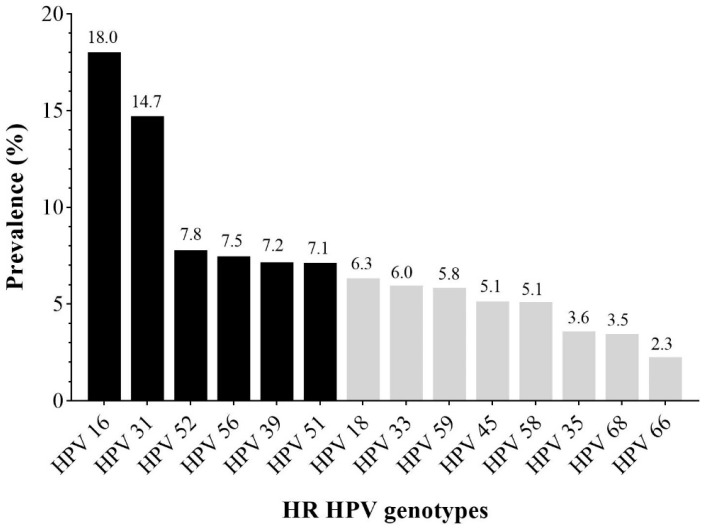
Genotype-specific distribution of detected HR HPVs. The black bars represent HR HPV genotypes with a prevalence of ≥7%, and the grey bars represent HR HPV genotypes with a prevalence of <7%. The genotype distribution of single HR HPV, double HR HPV, and infections caused by more than two HR HPVs is shown in Figure 3. HR HPV 16 is a unique genotype presented in a single HPV infection in more than half of cases (52.1%) (Figure 3).

In this diverse study, all HPV genotypes covered by the HPV test were identified (n = 7001) in the study population (4356 HR HPV-positive cervical samples). The prevalence of HR HPV genotypes is shown in Figure 2. The most prevalent HPV genotype is HPV 16, which makes up 18% (1262/7001) of the total HPV-detected genotypes in 29% (1262/4356) of HP-positive samples. HR HPV 31 takes second place, with 14.7% (1030/7001) of total HPV-detected genotypes in 23.6% (1030/4356) of HPV-DNA-positive samples. Thirdly, HR HPV 52 has 7.8% (545/7001) of total HPV-detected genotypes in 12.5% (545/4356) of HPV-DNA-positive samples. Fourthly, HR HPV 56 has 7.5% (523/7001) of total HPV-detected genotypes in 12% (523/4356) of HPV-DNA-positive samples. HR HPV 39 takes fifth place, with 7.2% (502/7001) of total HPV-detected genotypes in 11.5% (499/4356) of HPV-DNA-positive samples. Similarly, HR HPV 51 has 7.1% (499/7001) of HPV-detected genotypes in 11.5% (499/4356) of HPV-DNA-positive samples. The results show that those HR HPVs comprise 62.3% (4361/7001) of the detected genotypes, including multiple infections. Other HR HPV genotypes are less prevalent (2.3–6.3% of total HPV-detected genotypes) (Figure 2).

**Figure 3:**
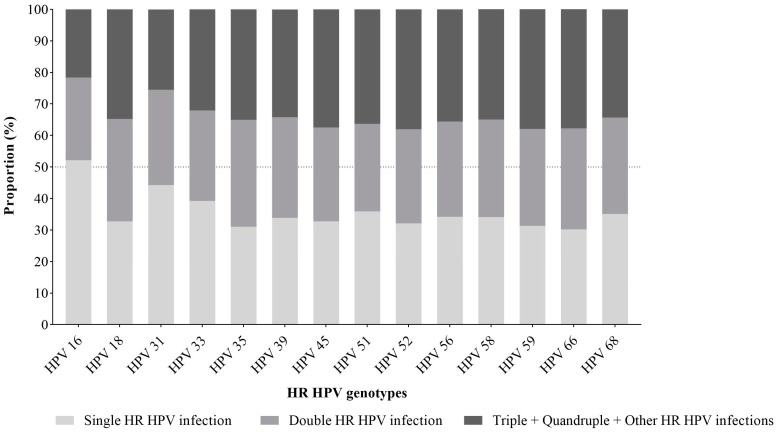
The genotype distribution of HR HPV infections among women from 2014 to 2023.

The most commonly detected HR HPV genotypes in double infection are listed in Table 1. The predominant co-infections were those with HR HPV 16 and 31, found in 7.9% (82/1040) of cases with double HPV infection. HR HPV 16 genotype associated with HPV 39 was registered with half as much participation (3.5%) (Table 1).

### 3.3. Age-Specific Prevalence of HR HPV Infection and Genotype Distribution

**Figure 4:**
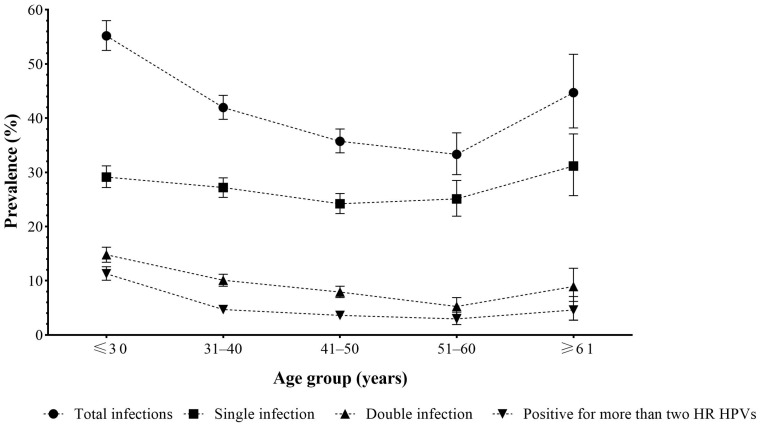
Prevalence of HR HPV infection in different age groups with 95% confidence interval bars.

The cases were classified into five groups according to the women’s ages (≤30, 31–40, 41–50, 51–60, and ≥61 years old) at the time of genotype testing to analyse the age-dependent trend of HPV prevalence. The total, single, double, and positive for more than two HR HPV genotypes infection rates of HR HPV in different age groups are shown in Figure 4. According to the total infections, as displayed, there was a significant difference in the prevalence across the various age groups (χ^2^ test, χ^2^ = 264.415, *p* < 0.001) (Supplementary Materials Table S1). The women younger than 31 years had the highest HR HPV prevalence, 55.2% (1546/2800, 95% CI: 52.5 to 58.0). There was a declining trend in the following age groups: 42.0% (1361/3242, 95% CI: 39.8 to 44.2), 35.7% (992/2775, 95% CI: 33.6 to 38.0), and 33.3% (292/876, 95% CI: 29.6 to 37.3) for age groups 31–40 years, 41–50 years, and 51–60 years, respectively. The second peak of the prevalence of total infection was noted for women older than 60 years, 44.7% (165/369, 95% CI: 38.2 to 51.8). Single infection was the most common infection type in all groups of age. The statistical significance of the prevalence of each pair of age groups is shown in Table S2 of Supplementary Materials. Double infection, and infections that comprise more than two HPV genotypes, had lower prevalence in all age groups, with the highest prevalence for those younger than 31 years, 14.8% (414/2800, 95% CI: 13.4 to 16.2) and 11.3% (316/2800, 95% CI: 10.1 to 12.6), respectively (Figure 4).

**Figure 5:**
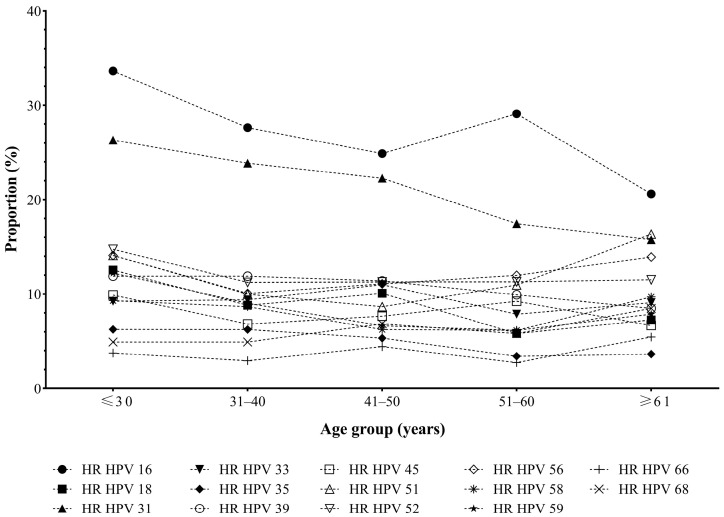
Distribution of HR HPV genotypes in different age groups among HPV-positive females.

The proportion of the positives for HR HPV genotypes in different age groups among HPV-positive women is shown in Figure 5. HR HPV 16 had the highest proportion in all age groups, with the peaks in the age groups of ≤30 years (33.6%) and 51–60 years (29.1%). The second most positive genotype is HR HPV 31, with a declining proportion across the age groups, from 26.3% (≤30 years) to 15.8% (≥61 years). Other HR HPV genotypes had a lower proportion in all age groups.

### 3.4. Cervical Cytology

A fraction of the total samples that were obtained from 2022 to 2023 (total of 2026 specimens) from women in Vojvodina were classified based on the Bethesda System 2014 into four categories by cytological criteria: NILM, ASCUS, LSIL, and HSIL. Further analyses were performed within the HR HPV-positive samples (1016 cervical samples). The molecular data of those samples were compared with the cytological results and age categories. A third of women, 34.3% (n = 348), had normal results, whereas 65.7% (n = 668) showed different cytological abnormalities. A total of 41.5% (n = 422) of the examined women had ASCUS; in 15.8% (n = 161), LSILs were found, whereas HSILs were detected in 8.4% (n = 85). The mean age of the patients was 37.7 years. Among the specimens, the number of Serbian women who were ≤30 years, 31–40 years, 41–50 years, 51–60 years, and ≥61 years old accounted for 32.2% (n = 327), 28.4% (n = 289), 27.0% (n = 274), 6.9% (n = 70), and 5.5% (n = 56) of the samples, respectively (Table 2).

The distribution of single and multiple HPV infections in different cytological groups is shown in Table 3. A statistically significant difference in the number of single and multiple HPV infections among the various cytological groups was observed (χ^2^ test; χ^2^ = 21.880; *p* < 0.0001). The prevalence of a single HPV infection increased with the severity of the cervical intraepithelial lesion and was the most prevalent in patients with HSIL cytological findings (76.5%). In the other groups of cytological findings, it was demonstrated in a lower percentage (49.7–66.7%). Multiple HR HPV infection was the least detected in HSIL (23.5%) compared to other cytological groups (33.3–50.3%) (Table 3).

The distribution of the detected HPVs concerning cytology is shown in Table 4. The prevalence rates of HR HPV 16 ranged from 23.9% in the group of NILM cytology to 57.6% in the HSIL group. Contrarily, the prevalence of HR HPV 31 was similar in the groups of NILM (18.4%), ASCUS (22.3%), and LSIL (26.7%), while it was lower in the group of HSIL (9.4%). Other HR HPV genotypes were present in all cytological groups in less than 19%. The statistically significant difference in the prevalence between the number of positive findings of following genotypes: HPV 16 (χ^2^ test; χ^2^ = 36.53; *p* < 0.0001), HPV 31 (χ^2^ test; χ^2^ = 11.95; *p* = 0.008), HPV 56 (χ^2^ test; χ^2^ = 9.55; *p* = 0.023), HPV 59 (χ^2^ test; χ^2^ = 9.29; *p* = 0.026), HPV 66 (χ^2^ test; χ^2^ = 13.54; *p* = 0.004), and HPV 68 (χ^2^ test; χ^2^ = 12.46; *p* = 0.006) was determined depending on the degree of severity of the cytological findings (Table 4).

A statistically significant difference was found in the number of women concerning the cytological findings and the age of the patients (χ^2^ test; χ^2^ = 31.98; *p* = 0.001) (Table 5). A statistically significant difference was determined regarding the cytological findings and the mean age of the patients, where the women diagnosed with HSIL were significantly older compared to the other groups (ANOVA; F = 5.583; *p* < 0.0008). The Bonferroni post hoc test determined that the women with HSIL were statistically significantly older than those with NILM (*p* < 0.001), ASCUS (*p* = 0.014), and LSIL (*p* = 0.001) (Table 5).

## 4. Discussion

Cervical cancer caused by persistent HPV infection is one of the most common malignant tumours in women. The role of HPV in the aetiology of human cancers of different locations has been studied for half a century, and, today, there is significant evidence that confirms the association of malignant diseases with this human carcinogen [3]. Therefore, the study of the prevalence and genotypic distribution of HPV is necessary for the development and implementation of cervical cancer screening programs and successful vaccination. It can be helpful to prospectively evaluate changes in HPV genotype distribution and the effectiveness of HPV vaccination [19]. Like some countries in the region, the Republic of Serbia is not subject to mandatory reporting of HPV infection, resulting in insufficient data on the incidence and prevalence of this virus. The available data are referred to in several studies [20,21]. This study aimed to obtain extensive information about the frequency and genotype distribution of the most prevalent HPV types among Serbian women. Our study tested 10,062 female patient samples from the north part of the Republic of Serbia (Vojvodina) for the HR HPV genotypes.

In our retrospective study, there were 4356 (43%) HPV-positive cervical samples (Figure 1). In part of them, with known cytological results (n = 1016), normal findings were registered in only one-third (34%), whereas 66% showed different degrees of cytological abnormalities (Table 2). These results are in line with our previous small-scale domestic studies related to HPV prevalence in Serbian women, which have reported that the presence of HR HPV infections ranges from 43 to 51%, and cytological abnormalities were registered in around two-thirds of examined women [20,21,22]. Additionally, it is in concordance with the results of the study of HPV prevalence in some European countries, which have reported a wide range prevalence of HPV in women with normal cytology (10%) and invasive cervical cancer (87%) [6]. In the countries of our region, a higher percentage of HPV infection was recorded in Croatia (59%) [19], and in the territory of Italy (53%) [23]. Lopicic et al. (2021) found nearly the same HPV infection rate (41%) in examined Montenegrin women [24]. Ursu et al. (2011), in Romanian women, proved the presence of HPV infection in 37% [25], while more recent data showed a lower prevalence (12%) [26]. A lower percentage of detected HPV infection (31%) compared to our results was observed among respondents in Greece [27] and Bulgaria (30%) [28], while 23% was determined in North Macedonia [29]. Observed differences in the prevalence of HPV infection among the examined women in these studies and the results of individual authors can be explained by the fact that the detected representation primarily depends on geographic areas, the size and construction of the sample, and the age of the examinee’s women, as well as choices, that is, the possibility of choosing the methods used for detection, which creates difficulties in comparison [4].

In this study, the most frequent HR HPV genotypes were HPV 16, 31, 52, 56, 39, and 51 (Figure 2), similar to those reported in several local studies from our country [20,21]. HPV 16 was registered in 29% of positive samples (1262/4356) as the most prevalent HR HPV genotype in each age group (Figure 5). The prevalence of this genotype is proportional to the degree of severity of the cervical lesion in the sample fraction with known cytology results (Table 4), which is consistent with studies conducted in our country [4,21] and other countries worldwide [3]. According to published data, it is estimated that the prevalence of HPV 16 in cervical intraepithelial neoplasia differs from other HR HPV genotypes, as well as that about 40% of high-grade cervical lesions are positive for it. Additionally, the progression of HPV infection to high-grade lesions and more at ten years occurs in about 15% of cases, which is higher than other HR HPV. Therefore, this HPV genotype-dependent carcinogenesis may be related to different expression patterns [30].

Although observed globally, HR HPV 18 is the second most common genotype in cervical cancer. Results of several studies suggest that this HR HPV is detected among the top five HPVs [19,25,31]. However, infections caused by it are not among the most common in Serbian women (Figure 2), which supports the fact that HPV genotype distributions depend on local diversity. In this regard, a recent 11-year retrospective study from Cypres reported a similar prevalence (6.6%) of HR HPV 18 [32]. A linear correlation with the degree of severity of cervical abnormalities was expressed for HPV 16, but not for HPV 18, as shown in this study (Table 4) and other previously published data [3]. This occurrence can be explained by the different mechanisms of carcinogenesis, which depend on the HPV genotype, the effect of viral load, and its physical state [33]. This result is in line with the presentation of this genotype by cytological groups worldwide, where its participation is significantly lower in normal cytological findings and precancerous cervical lesions compared to cancer [3].

This study’s second most common genotype was HR HPV 31 (Figure 2), which has been observed in research from other European studies [19,31,34] including comprehensive results of a meta-analysis conducted on five continents [35]. According to the publication of the International Agency for Research on Cancer (2023) [3], which states the comparison of the ten most frequent HPV oncogenic genotypes in the world among women with and without cervical lesions, this genotype has the opposite trend of HPV 18. HR HPV 31 is among the first four most frequently represented HPVs in normal cytology and precancerous lesions, while its participation in cancers is lower [3]. This trend is similar to ours, where the participation of this genotype decreases with the severity of the lesion (Table 4). In light of these considerations, the oncogene activity, precisely, the expression tendency of the E6/E7 mRNA HR HPV 31, was detected at a lower level in HSIL than in other cytological groups [18].

It is also worth noting that HPV 52, 56, 51, and 39 are registered in approximately the same percentage (7.1–7.8%) and ranked in the top six HPV genotypes detected in our study (Figure 2). Previous studies have reported similar observations of HPV 51 that referred to certain European countries [36,37,38], including our region [20,21]. Although non-vaccinal genotypes (HPV 35, 39, 51, 56, 59, and 68) are currently thought to be causative in less than 10% of cervical cancers worldwide [39], the high frequency in our region and absence of these genotypes (HPV 39, 51, and 56) in the vaccine suggest that a detailed characterisation of the mechanism of their oncogenic action could be the subject of future research.

According to the infection patterns in the current research, single HR HPV infections are dominant (62%) and increase with the severity of the cervical intraepithelial lesion, followed by double HR HPV infections (24%) (Figure 1, Table 3). Regarding age, the presence of a single HPV infection was consistent with the total participation, while the presence of double infections and HPV infections positive for more than two HR HPV genotypes decreased with age (Figure 4). Despite their observation, the clinical and virological role of multiple HR HPV infections is still unclear and remains debatable. Although, in some studies, co-infections have been associated with a greater risk of cervical cancer, studies objecting to multiple HPV infections suggest that their presence does not affect the course of infection (for references see [40]). On that note, Bruno et al. (2020) [40] state that the presence of multiple HPV infections is associated only with early stages of cervical lesions (CIN1-CIN2) while emphasising the absence of their synergic action in carcinogenesis.

If the prevalence of multiple infections is observed concerning individual HPV genotypes, it can be noted that, in our research, HPV 16 is the most frequently represented genotype in female co-infections. Its participation in combination with HPV 31 occurs in 8% of registered double infections, while this genotype, associated with HPV 39, is registered with half as much participation (Table 1). Different patterns of combination are observed in different regions of the world, leading to an increase in the observation of these infections in recent years. Our results showed a tendency for specific genotypes to appear simultaneously, which agrees with the results of other studies. The highest prevalence of single (Figure 3), and, therefore, combined (Table 1), occurrence of the HPV 16 genotype is also observed in the study by Hajia and Sohrabi (2018) [41], but this time in combination with HR HPV 53, 31, and 52. It was found that simultaneous detection of any two genotypes that belong to Alpha 9 was associated with a significantly increased risk for high-grade cervical lesions [42], while the study by Bruno et al. (2020) [40] states that only the occurrence of specific combination, such as HR HPV 16 and 31 and HR HPV 16 and 18, may have a clinically significant effect on the formation of high-grade lesions.

Two patterns were observed for age-specific HPV prevalence around the world. The appearance of a bimodal curve shows that the primary prevalence peak is among women up to 25 years of age, followed by a plateau of prevalence during the middle age of life, followed by another rebound in women after 45 years. However, other authors state the existence of a pattern in which the initial peak is observed at younger ages, followed by a gradual decline at older ages [43]. Regarding age, the results of this study observed a bimodal distribution of HPV infection (Figure 4). There was a significant difference in the prevalence of total HR HPV infections across the various age groups (χ^2^ test, χ^2^ = 264.415, *p* < 0.001) (Supplementary Materials Table S1). More precisely, the first peak was recorded under the age of 31 years, which was in line with the previous study [44]; then, it decreased slightly and maintained a plateau in the middle-age group of women (31–60 years), followed by a second peak in the group after 61 years (Figure 4). Age at first sexual activity corresponds with the first peak [4]. Most HPV infections in young women will be transient and cleared by the immune system [1], consistent with a plateau of prevalence during middle age after the first peak. The reason for the appearance of the second peak of HPV infection can be explained by the reactivation of latent infection that can occur at this age [45], which can reflect an impaired immune response associated with hormonal changes at the menopausal transition [46] as well as the increased susceptibility to acquiring a new HPV infection in women with previous high-grade lesions [47]. In this regard, recent data highlight that HPV vaccines are indicated in persons older than nine years, with no upper age limit. Age-independent catch-up vaccination may be considered in populations at increased risk of HPV-related disease and post-treatment relapse due to immunocompromise [48]. According to our results, HR HPV 16 is the most frequently detected genotype in the group of female patients over the age of 50 years (Figure 5). In addition, the results indicated that the highest percentage of high-grade lesions was recorded in the group of patients over 50 years of age (Table 5), which is in accordance with the findings that the risk from the progression of an existing high-grade lesion increases with age. It is stated that, in women over 50 years of age, the risk of cervical cancer increases seven times [47].

To prevent HPV infection in young people and, consequently, HPV-related cancer, HPV vaccination is the most effective tool, particularly when it is administrated before the initiation of sexual activity [11]. The importance of the use of vaccines in the prevention of cervical cancer has been confirmed and has been included in many national immunisation strategies. Over half of World Health Organization (WHO) member countries (63%) have introduced HPV vaccination partially or nationwide [49]. According to our data, high-risk genotypes HPV 16 and 18 were detected in 24% of infections, and, when considering the presence of vaccine genotypes HPV 16, 18, 31, 33, 45, 52, and 58 in the examined sample, account for 63% of infections (Figure 2). Based on the obtained results related to the distribution of oncogenic HPV in women of Vojvodina, it can be assumed that the application of the 9vHPV vaccine is more appropriate for Serbian women. This estimate is lower but still incalculably valuable in comparison to studies that found 9vHPV vaccines can protect against HPV with an effectiveness of 91% in Europe, 92% in Africa and North America, and 88% in Asia (reviewed in [11]).

As the 9vHPV vaccine was introduced in the National immunisation program in Serbia and has been applied since 2022, the HPV vaccination rate in our country has not yet been precisely defined. The uptake rate of HPV vaccination varies between countries and depends on many factors. According to WHO, vaccination hesitancy is one of the biggest health threats [50]. Considering that the HPV vaccination program in our country is recommended and refers to children and adolescents (aged 9–19 years) whose vaccination requires parental consent (aged 9–14 years), the success of the HPV vaccination program depends on the parents’ attitudes. Many studies have determined which factors may influence parents’ decisions and motivation [51]. According to the findings of Štrbac et al. (2023) [52], the strongest motive for HPV vaccination in Serbia was the “Recommendation from a paediatrician”, which agrees with studies that named this motive the most important predictor of HPV immunisation [53].

This study has several limitations. Firstly, the examined population of women is limited, consisting of a portion of patients who cannot represent the total population of Serbian women. Additionally, the number of positive HPV infection findings that can be correlated with the degree of cervical lesions is limited and refers to the fraction of the total sample. Third, vaccination coverage data are unavailable, so assessing the vaccine’s impact is impossible. Next, data for genotypes 66 and 68 were not obtained during the years 2014, 2019, 2020, and 2021 because these two genotypes were not included in the diagnostic kit used for those years. The COVID-19 pandemic has decreased the number of visits to gynaecologists during the lockdown, so it is necessary to continue the follow-up of this type of research.

## 5. Conclusions

In conclusion, this research revealed a high overall prevalence of HPV infection (43%) in this study’s examined female population of Serbia over ten years. The most prevalent oncogenic HPV genotypes were HPV 16, 31, 52, 56, 39, and 51, approximately two-thirds of the total registered HPV genotypes. It is important to note that non-vaccinated HPV genotypes (HPV 39, 51, and 56) were a non-negligible part of the examined sample, which is why applying cervical screening is very significant. Concerning age, the results of this study observed a bimodal distribution of HPV infection. The highest prevalence was recorded in women under 31 and was slightly lower after 60 years, emphasising the expediency of administering the vaccine at a younger age and the importance of cervical screening tests at all observed ages. Considering the lack of data on the prevalence of HPV infection in the population of Serbian women, these data provide insight into the current state. They can serve as a basis for subsequent monitoring of genotypic distribution, which is particularly significant considering that the cervical cancer mortality rate in Serbia is one of the highest among European countries [3].

## Figures and Tables

**Table 1:** HR HPV distribution in double infections.

HR HPV Genotypes	Double Infection		
	n	%	% of All Infected Women
HPV 16, HPV 31	82	7.9 (82/1040)	1.9 (82/4356)
HPV 16, HPV 39	36	3.5 (35/1040)	0.8 (36/4356)
HPV 31, HPV 52	32	3.1 (32/1040)	0.7 (32/4356)
HPV 16, HPV 56	31	3.0 (31/1040)	0.7 (31/4356)
HPV 31, HPV 39	30	2.9 (30/1040)	0.7 (30/4356)
HPV 18, HPV 39	28	2.7 (28/1040)	0.6 (28/4356)
HPV 16, HPV 52	26	2.5 (26/1040)	0.6 (26/4356)
HPV 16, HPV 51	25	2.4 (25/1040)	0.6 (25/4356)
HPV 16, HPV 18	24	2.3 (24/1040)	0.6 (24/4356)
HPV 16, HPV 59	24	2.3 (24/1040)	0.6 (24/4356)
HPV 18, HPV 31	24	2.3 (24/1040)	0.6 (24/4356)
HPV 31, HPV 56	23	2.2 (23/1040)	0.5 (23/4356)
Other double infections:	655	63.0 (655/1040)	15 (655/4356)
Total:	1040	100 (1040/1040)	23.9 (1040/4356)

HR HPV—high-risk human papillomavirus; n—number of HR HPV-positive women with double infections.

**Table 2:** Cervical cytology and age of female patients.

HR HPV-Positive Women *	n (%)
Cytology	
NILM	348 (34.3)
ASCUS	422 (41.5)
LSIL	161 (15.8)
HSIL	85 (8.4)
Total	1016 (100)
Age	
≤30	327 (32.2)
31–40	289 (28.4)
41–50	274 (27.0)
51–60	70 (6.9)
≥61	56 (5.5)
Mean age (years, SD))	37.7 (12.1)

n—number of positive cases; * HR HPV-positive women with known cytology results. SD—standard deviation; NILM—negative for an intraepithelial lesion or malignancy; ASCUS—atypical squamous cells of unknown significance; LSIL—low-grade squamous intraepithelial lesions; HSIL—high-grade squamous intraepithelial lesions.

**Table 3:** Distribution of single and multiple HPV infections according to cytological findings.

HR HPV Infection	NILM	ASCUS	LSIL	HSIL	χ^2^	*p*
	n (%)	n (%)	n (%)	n (%)		
Single	232 (66.7)	253 (60.0)	80 (49.7)	65 (76.5)	21.880	0.000 ***
Multiple	116 (33.3)	169 (40.0)	81 (50.3)	20 (23.5)		
Total	348 (100)	422 (100)	161 (100)	85 (100)		

n—number of positive cases; *** *p* < 0.001. HR HPV—high-risk human papillomavirus; NILM—negative for an intraepithelial lesion or malignancy; ASCUS—atypical squamous cells of unknown significance; LSIL—low-grade squamous intraepithelial lesions; HSIL—high-grade squamous intraepithelial lesions.

**Table 4:** Distribution of the HR HPVs according to cytology.

HR HPV		Cytology				χ^2^	*p*
		NILM	ASCUS	LSIL	HSIL		
		n (%)	n (%)	n (%)	n (%)		
HPV 16	+	83 (23.9)	136 (32.2)	51 (31.7)	49 (57.6)	36.530	0.000 ***
	−	265 (76.1)	286 (67.8)	110 (68.3)	36 (42.4)		
HPV 18	+	42 (12.1)	34 (8.1)	21 (13.0)	8 (9.4)	4.845	0.184
	−	306 (87.9)	388 (91.9)	140 (87.0)	77 (90.6)		
HPV 31	+	64 (18.4)	94 (22.3)	43 (26.7)	8 (9.4)	11.950	0.008 **
	−	284 (81.6)	328 (77.7)	118 (73.3)	77 (90.6)		
HPV 33	+	25 (7.2)	49 (11.6)	12 (7.5)	9 (10.6)	5.309	0.151
	−	323 (92.8)	373 (88.4)	149 (92.5)	76 (89.4)		
HPV 35	+	13 (3.7)	30 (7.1)	12 (7.5)	3 (3.5)	5.715	0.126
	−	335 (96.3)	392 (92.9)	149 (92.5)	82 (96.5)		
HPV 39	+	24 (6.9)	39 (9.2)	15 (9.3)	4 (4.7)	3.061	0.382
	−	324 (93.1)	383 (90.8)	146 (90.7)	81 (95.3)		
HPV 45	+	26 (7.5)	31 (7.3)	12 (7.5)	4 (4.7)	0.860	0.835
	−	322 (92.5)	391 (92.7)	149 (92.5)	81 (95.3)		
HPV 51	+	42 (12.1)	53 (12.6)	24 (14.9)	8 (9.4)	1.654	0.647
	−	306 (87.9)	369 (87.4)	137 (85.1)	77 (90.6)		
HPV 52	+	39 (11.2)	61 (14.5)	15 (9.3)	7 (8.2)	4.851	0.183
	−	309 (88.8)	361 (85.5)	146 (90.7)	78 (91.8)		
HPV 56	+	34 (9.8)	50 (11.8)	30 (18.6)	7 (8.2)	9.552	0.023 *
	−	314 (90.2)	372 (88.2)	131 (81.4)	78 (91.8)		
HPV 58	+	26 (7.5)	30 (7.1)	14 (8.7)	3 (3.5)	2.301	0.512
	−	322 (92.5)	392 (92.9)	147 (91.3)	82 (96.5)		
HPV 59	+	40 (11.5)	37 (8.8)	18 (11.2)	1 (1.2)	9.293	0.026 *
	−	308 (88.5)	385 (91.2)	143 (88.8)	84 (98.8)		
HPV 66	+	37 (10.6)	24 (5.7)	23 (14.3)	5 (5.9)	13.540	0.004 **
	−	311 (89.4)	398 (94.3)	138 (85.7)	80 (94.1)		
HPV 68	+	50 (14.4)	56 (13.3)	16 (9.9)	1 (1.2)	12.460	0.006 **
	−	298 (85.6)	366 (86.7)	145 (90.1)	84 (98.8)		
Total		348 (100)	422 (100)	161 (100)	85 (100)		

n—number of positive cases; * *p* < 0.05, ** *p* < 0.01, *** *p* < 0.001. HR HPV—high-risk human papillomavirus; NILM—negative for an intraepithelial lesion or malignancy; ASCUS—atypical squamous cells of unknown significance; LSIL—low-grade squamous intraepithelial lesions; HSIL—high-grade squamous intraepithelial lesions.

**Table 5:** Age-specific distribution of female patients with different cytological groups.

Cytology	Age Group (Years)					Total n (%)	χ^2^	*p*	Mean Age (Years, (SD))	F	*p*
	≤30	31–40	41–50	51–60	≥61						
	n (%)	n (%)	n (%)	n (%)	n (%)						
NILM	125 (38.2)	94 (32.5)	94 (34.3)	17 (24.3)	18 (32.1)	348 (34.3)	31.98	0.001 **	36.6 (11.9)	5.583	0.000 ***
ASCUS	130 (39.8)	118 (40.8)	121 (44.2)	31 (44.3)	22 (39.3)	422 (41.5)			38.1 (11.8)		0.014 *
LSIL	55 (16.8)	45 (15.6)	46 (16.8)	9 (12.9)	6 (10.7)	161 (15.8)			36.5 (11.6)		0.001 **
HSIL	17 (5.2)	32 (11.1)	13 (4.7)	13 (18.6)	10 (17.9)	85 (8.4)			42.2 (14.4)		-
Total	327 (32.2)	289 (28.4)	274 (27.0)	70 (6.9)	56 (5.5)	1016 (100)					

n—number of positive cases; SD—standard deviation; F—F-test in analyses of variance (ANOVA); * *p* < 0.05, ** *p* < 0.01, *** *p* < 0.001. NILM—negative for an intraepithelial lesion or malignancy; ASCUS—atypical squamous cells of unknown significance; LSIL—low-grade squamous intraepithelial lesions; HSIL—high-grade squamous intraepithelial lesions.

## Data Availability

The data that support the findings of this study are available from the corresponding author upon reasonable request.

## Supplementary Materials

The following supporting information can be downloaded at the following location: https://www.mdpi.com/article/10.3390/medicina60060922/s1, Table S1: Prevalence of total HR HPV infections in different age groups; Table S2: The statistical significance of the prevalence of the total HR HPV infections (shown in Table S1), of each pair of age groups.
