# Supplementary material for: Prevalence of Carcinogenic Genotypes of HPV-Infected Women in a Ten-Year Period (2014–2023) in Vojvodina, Serbia
Source: Medicina (Kaunas). 2024 Jun 1;60(6):922. doi: 10.3390/medicina60060922 (PMC11205824; doi:10.3390/medicina60060922)
Supplement: Supplementary file 1 [file medicina-60-00922-s001.zip › medicina-2986615-supplementary.pdf]

Supplementary Material

Prevalence of Carcinogenic Genotypes of HPV-infected Women in a Ten-Year Period (2014–2023) in Vojvodina, Serbia

Natasa Nikolic[1,2], Branka Basica[1*], Mirjana Strbac[1], Lidija Terzic[1], Aleksandra Patic[1,2], Gordana Kovacevic[1,2], Radmila Velicki[1,2], Dusan Petrovic[1], Aljosa Mandic[2,3], and Vladimir Petrovic[1,2]

[1] Institute of Public Health of Vojvodina, 21000 Novi Sad, Serbia; natasa.nikolic@mf.uns.ac.rs; mirjana.strbac@izjzv.org.rs; lidija.terzic@izjzv.org.rs; aleksandra.patic@mf.uns.ac.rs; gordana.kovacevic@izjzv.org.rs; radmila.velicki@mf.uns.ac.rs; dusan.petrovic@izjzv.org.rs; vladimir.petrovic@mf.uns.ac.rs
[2] Faculty of Medicine, University of Novi Sad, 21000 Novi Sad, Serbia; aljosa.mandic@mf.uns.ac.rs
[3] Clinic for Oncological Surgery, Oncology Institute of Vojvodina, 21204 Sremska Kamenica, Serbia
[*] Correspondence: branka.basica@izjzv.org.rs

Table S1. Prevalence of total HR HPV infections in different age groups.

Total HR HPV infections	Age Group (Years)					χ^2	*p*
	≤ 30	31–40	41–50	51–60	≥ 61		
	n (%)	n (%)	n (%)	n (%)	n (%)		
**Positive**	1,546 (55.2)	1,361 (42.0)	992 (35.7)	292 (33.3)	165 (44.7)	264,416	0.000***
**Negative**	1,254 (44.8)	1,881 (58.0)	1,783 (64.3)	584 (66.7)	204 (55.3)		
**Total:**	2,800 (100)	3,242 (100)	2,775 (100)	876 (100)	369 (100)		

n – number of positive cases; ***$p < 0.001$. HR HPV—high-risk human papillomavirus.

Table S2. The statistical significance of the prevalence of the total HR HPV infections (shown in Table S1), of each pair of age groups.

Total HR HPV infections	Age Group (Years)				
Age Group (Years)	≤ 30	31–40	41–50	51–60	≥ 61
	χ^2 (*p*)	χ^2 (*p*)	χ^2 (*p*)	χ^2 (*p*)	χ^2 (*p*)
**≤ 30**	-	105,403 (***)	212.964 (***)	127,785 (***)	14,467 (***)
**31–40**	105,403 (***)	-	24,390 (***)	21,460 (***)	1,016 (0,314)
**41–50**	212.964 (***)	24,390 (***)	-	1,702 (0,192)	11,262 (**)
**51–60**	127,785 (***)	21,460 (***)	1,702 (0,192)	-	14,478 (***)
**≥ 61**	14,467 (***)	1,016 (0,314)	11,262 (**)	14,478 (***)	-

** $p < 0.01$; ***$p < 0.001$. HR HPV—high-risk human papillomavirus.
